# Impact of interleukin-6 on hypoxia-induced pulmonary hypertension and lung inflammation in mice

**DOI:** 10.1186/1465-9921-10-6

**Published:** 2009-01-27

**Authors:** Laurent Savale, Ly Tu, Dominique Rideau, Mohamed Izziki, Bernard Maitre, Serge Adnot, Saadia Eddahibi

**Affiliations:** 1 INSERM U841, Université Paris XII, F94010 Créteil, France; 2 AP-HP, Hôpital Henri Mondor, Service de Physiologie Explorations Fonctionnelles, F94010 Créteil, France; 3 AP-HP, Hôpital Henri Mondor, Unité de Pneumologie, F94010 Créteil, France

## Abstract

**Background:**

Inflammation may contribute to the pathogenesis of various forms of pulmonary hypertension (PH). Recent studies in patients with idiopathic PH or PH associated with underlying diseases suggest a role for interleukin-6 (IL-6).

**Methods:**

To determine whether endogenous IL-6 contributes to mediate hypoxic PH and lung inflammation, we studied IL-6-deficient (IL-6^-/-^) and wild-type (IL-6^+/+^) mice exposed to hypoxia for 2 weeks.

**Results:**

Right ventricular systolic pressure, right ventricle hypertrophy, and the number and media thickness of muscular pulmonary vessels were decreased in IL-6^-/- ^mice compared to wild-type controls after 2 weeks' hypoxia, although the pressure response to acute hypoxia was similar in IL-6^+/+ ^and IL-6^-/- ^mice. Hypoxia exposure of IL-6^+/+ ^mice led to marked increases in IL-6 mRNA and protein levels within the first week, with positive IL-6 immunostaining in the pulmonary vessel walls. Lung IL-6 receptor and gp 130 (the IL-6 signal transducer) mRNA levels increased after 1 and 2 weeks' hypoxia. In vitro studies of cultured human pulmonary-artery smooth-muscle-cells (PA-SMCs) and microvascular endothelial cells revealed prominent synthesis of IL-6 by PA-SMCs, with further stimulation by hypoxia. IL-6 also markedly stimulated PA-SMC migration without affecting proliferation. Hypoxic IL-6^-/- ^mice showed less inflammatory cell recruitment in the lungs, compared to hypoxic wild-type mice, as assessed by lung protein levels and immunostaining for the specific macrophage marker F4/80, with no difference in lung expression of adhesion molecules or cytokines.

**Conclusion:**

These data suggest that IL-6 may be actively involved in hypoxia-induced lung inflammation and pulmonary vascular remodeling in mice.

## Background

Inflammation is now recognized as a potential contributor to the pathogenesis of both idiopathic pulmonary hypertension (PH) and PH associated with underlying diseases [1,2]. Perivascular inflammatory cell infiltrates are found in lungs from patients with PH or chronic obstructive pulmonary disease (COPD) [2,3]. Compared to healthy controls, patients with idiopathic or associated PH exhibit higher circulating levels and pulmonary expression of various inflammatory cytokines and chemokines including interleukin-1beta (IL-1β), IL-6, monocyte chemoattractant protein (MCP-1), RANTES, and fractalkine [4-10]. In recent studies of patients with COPD, we found that pulmonary artery pressure correlated positively with the circulating levels of two cytokines, namely, IL-6 and MCP-1 [11]. Moreover, a close relationship was found between the G(-174)C polymorphism of the IL-6 gene and the severity of PH in our patients with COPD. This polymorphism influences the levels of circulating IL-6, suggesting a causal role for high circulating IL-6 levels in the pathogenesis of PH in patients with COPD.

IL-6 is a multifunctional proinflammatory cytokine that is linked to a number of disorders including systemic and pulmonary vascular diseases [12]. IL-6 is now considered a major biomarker for cardiovascular risk and the main stimulant for hepatic production of C-reactive protein, a compound widely used as a biomarker for atherosclerosis [13]. A role for IL-6 in the pathogenesis of various forms of PH was suggested by clinical and experimental studies. Elevated serum IL-6 concentrations have been reported in patients with idiopathic PH or PH associated with inflammatory diseases such as scleroderma, lupus, and POEMS syndrome [4,14-16], although other studies did not confirm these findings in patients with idiopathic PH or connective tissue disease [17]. Increased IL-6 levels have been documented in lungs from animals exposed to chronic hypoxia [18]. IL-6 elevation reported during acute hypoxia was suggested to affect lung vascular permeability and the early inflammatory response to hypoxia [19,20]. The recent finding that exogenously administered IL-6 aggravates the development of PH in mice exposed to chronic hypoxia points to a role for IL-6 in pulmonary vascular remodeling [21]. Infusion of IL-6 has also been shown to cause pulmonary vascular thrombosis and vessel occlusion, indicating prothrombotic and proinflammatory interactions with circulating cells [22,23]. More recently, IL-6 overexpressing transgenic mice have been shown to develop spontaneous pulmonary vascular remodeling and PH [24]. However, the influence of physiological levels of endogenous IL-6 on the development of PH remains unknown. Thus, it is unclear whether IL-6 contributes to the process of pulmonary vascular remodeling during exposure to chronic hypoxia and how it affects the pulmonary vasculature.

The purpose of this study was to investigate whether IL-6 deficiency affected the development of pulmonary vascular remodeling and PH during chronic hypoxia. We used mice with targeted disruption of the IL-6 gene to investigate PH development and lung macrophage infiltration during exposure to chronic hypoxia [25].

## Materials and methods

### Mice

Mice lacking IL-6 (IL-6^-/-^) were generated by homologous recombination on the C57Bl/6 and IL-6^-/- ^genetic background [25]. The wild-type IL-6^+/+ ^and mutant homozygous IL-6^-/- ^mice used in this study were male littermates obtained by breeding heterozygous mutants. Genotypes were determined by polymerase chain reaction (PCR) analysis of tail biopsies to detect either the presence of the inactivating neomycin gene and/or the presence of the disrupted (IL-6^-/- ^mice) or intact (IL-6^+/+ ^mice) IL-6 gene. Mice aged 8–10 weeks were randomly allocated to room air or chronic hypoxia. All animal care and procedures were in accordance with institutional guidelines.

### Hemodynamic response of normoxic mice to acute hypoxia

Mice were anesthetized with intraperitoneal ketamine (6 mg/100 g) and xylazine (1 mg/100 g). The trachea was cannulated, and the lungs were ventilated with room air at a tidal volume of 0.2 ml and a rate of 90 breaths per minute. A 26-gauge needle was then introduced percutaneously into the right ventricle via the subxyphoid approach. Right ventricular systolic pressure (RVSP) was measured. RVSP and heart rate were recorded first while the animal was ventilated with room air then after 5 min of ventilation with the hypoxic gas mixture (8% O_2_, 92% N_2_). The heart rate under these conditions was between 300 and 500 bpm. If the heart rate fell below 300 bpm, measurements were excluded from analysis.

### Exposure to chronic hypoxia

Mice were exposed to chronic hypoxia (10% O_2_) in a ventilated chamber (500-L volume; Flufrance, Cachan, France) as described previously [26]. The hypoxic environment was established by flushing the chamber with a mixture of room air and nitrogen, and the gas was recirculated. The chamber environment was monitored using an oxygen analyzer. Carbon dioxide was removed by soda lime granules, and excess humidity was prevented by cooling of the recirculation circuit. Normoxic mice were kept in a similar chamber flushed with normoxic gas, in the same room and with the same light-dark cycle.

### Assessment of pulmonary hypertension

Mice exposed previously to hypoxia or room air for 1 day, 1 week, or 2 weeks were anaesthetized. After incision of the abdomen, a 26-gauge needle connected to a pressure transducer was inserted into the right ventricle through the diaphragm, and RVSP was recorded immediately. Then, the thorax was opened and the lungs and heart were removed. The right ventricle (RV) was dissected from the left ventricle plus septum (LV+S), and these dissected samples were weighed for determination of Fulton's index (RV/LV+S). The lungs were fixed by intratracheal infusion of 4% aqueous buffered formalin. A midsagittal slice of the right lung was processed for paraffin embedding. Sections 5 μm in thickness were cut and stained with hematoxylin-phloxine-saffron for examination by light microscopy. In each mouse, a total of 20 to 30 intraacinar vessels with diameters in the 50–200 μm range, accompanying either alveolar ducts or alveoli, were examined by an observer who was blinded to the genotype. Each vessel was categorized as nonmuscular (no evidence of vessel wall muscularization), partially muscular (smooth muscle cells [SMCs] identifiable in less than three-fourths of the vessel circumference), or fully muscular (SMCs in more than three-fourths of the vessel circumference). The percentage of pulmonary vessels in each muscularization category was determined by dividing the number of vessels in that category by the total number counted in the relevant group of animals. For fully muscular vessels, video images were obtained and arterial diameters were measured using image-analysis software. Percent wall thickness was then calculated as the diameter of the external elastic lamina minus the diameter of the internal lamina divided by the diameter of the external elastic lamina.

### Total RNA isolation

Total RNA was extracted from the lungs using the Qiagen RNeasy Mini kit (QIAGEN SA, Courtaboeuf, France) according to the manufacturer's instructions and estimated using optical density measurements (260- to 280-nm absorbance ratio). The RNA concentration was determined using standard spectrophotometric techniques, and RNA integrity was assessed by visual inspection of ethidium bromide-stained denaturing agarose gels.

### cDNA preparation and Real-Time Quantitative Polymerase Chain Reaction

First-strand cDNA synthesis was carried out using the SuperScript II Reverse Transcriptase System (Life Technologies. Inc, Gaithersburg, MD). A mixture containing 2 μg total RNA, 2 μL deoxynucleotide triphosphate mix (10 nmol/L), and 100 ng random primers in a total volume of 12 μL was incubated for 5 minutes at 65°C and chilled on ice. Then, 4 μL of 1^st ^Strand Buffer, 2 μL of DTT (0.1 mol/L), and 40 U of ribonuclease inhibitor (RNAse-Out, Invitrogen, Carlsbad, CA) were added to the samples, which were then heated at 25°C for 2 minutes. After addition of 1 μL SuperScript reverse transcriptase II (200 U/μL), the mixture was incubated for 10 minutes at 25°C, 50 minutes at 42°C, and 15 minutes at 70°C. The cDNA was diluted 1:40 for use in the real-time quantitative polymerase chain reaction. Amplification was performed in duplicate using the ABI Prism 7000 system (Applied Biosystems. Foster City, CA). PCR primers were designed using Primer Express Software (Applied Biosystems). To avoid inappropriate amplification of residual genomic DNA, intron-spanning primers were selected and internal control 18S rRNA primers provided. Primers used for detecting RNAs for IL-6, sIL-6-R, gp130, ET-1, MCP-1, ICAM, and VCAM in the lungs are listed in table 1. For each sample, the amplification reaction was performed in duplicate using SyberGreen mix and specific primers. Signal detection and analysis of results were performed using ABI-Prism 7000 sequence detection software (Applied Biosystems). The relative expression level of the genes of interest was computed relative to the mRNA expression level of the internal standard, r18S, as follows: relative mRNA = 1/2(Ct_gene of interest_-Ct_r18S_).

**Table 1:** Forward and reverse primers used in the study.

	**Forward 5'-3'**	**Reverse 5'-3'**
IL-6 mouse	CTCTGGGAAATCGTGGAAATG	AAGTGCATCATCGTTGTTCATACA
IL-6R mouse	GACTATTTATGCTCCCTGAATGATCA	ACTCACAGATGGCGTTGACAAG
gp-130 mouse	CAATTTTGACCCCGTGGATAA	GATAATTCTTCTGAGTTGGTCACTGA
MCP-1 mouse	TCTGGGCCTGCTGTTCACA	GGATCATCTTGCTGGTGAATGA
ET-1 mouse	TGGACAAGCAGTGTGTCTACTTCTC	GACGCGCTCGGGAGTGT
ICAM mouse	CCGCTTCCGCTACCATCA	CAGGCTGGCAGAGGTCTCA
VCAM mouse	ACGGTACTTTGGATACTGTTTGCA	GGCCATGGAGTCACCGATT

### Protein extraction and ELISA

Proteins were extracted from 100-mg snap-frozen tissue samples by homogenization in an appropriate amount of homogenizing Rippa Buffer containing protease inhibitors. The homogenates were centrifuged at 4°C and the supernatants were collected. IL-6 protein expression was assessed in homogenates of total lungs from IL-6^+/+ ^mice after exposure to 24 hours, 1 week, or 2 weeks of hypoxia and in normoxia. In brief, 50 μl of lung homogenate was incubated with 50 μl of assay diluent for 2 h at room temperature in a 96-well plate coated with a monoclonal antibody against IL-6. After three washes, a conjugate of polyclonal IL-6 antibody and horseradish peroxidase was added and incubated for 2 h at room temperature. After addition of a color reagent, absorbance was measured at 450 nm in a ThermoMax microplate reader. Results were normalized for the protein concentration previously determined using the Bradford method. For standardization, serial dilutions of recombinant mouse IL-6 were assayed at the same time.

### Lung immunohistochemical labeling of IL-6 and macrophages

Paraffin sections of lung specimens, each 5 mm in thickness, were mounted on Superfrost Plus slides (Fisher Scientific, Illkirch, France). For IL-6 and macrophage immunostaining, the slides were dewaxed in 100% toluene, and the sections were then rehydrated by immersion in decreasing ethanol concentrations (100%, 95%, and 70%) then in distilled water. Endogenous peroxidase activity was blocked with H_2_O_2 _in methanol (0.3% vol/vol) for 10 minutes. After three washes with PBS, the sections were preincubated in PBS supplemented with 3% (vol/vol) bovine serum albumin for 30 minutes then incubated overnight at 4°C with polyclonal goat anti-IL-6 (Santa Cruz Biotechnology, Santa Cruz, CA) or rat antibodies to the specific mouse macrophage marker F4/80 (AbD Serotec, Kidlington, Oxford, England), each diluted 1:500 in PBS containing 0.02% bovine serum albumin. The sections were exposed for 1 hour to biotin-labeled universal secondary antibodies (Dako, Trappes, France) in the same buffer then to streptavidin biotin horseradish peroxidase solution. Peroxidase staining was carried out using 3,3'-diaminobenzidine tetrahydrochloride dihydrate (DAB, Sigma, St Louis, MO) and hydrogen peroxide. Finally, the sections were stained with hematoxylin and eosin.

### F4/80 Western Blotting

After determination of the protein concentration in total lung homogenates using the Bradford method, 30 μg of protein from each lung sample was resuspended in 3× Laemmli buffer, boiled for 5 min, and separated on 8% acrylamide gels by electrophoresis. Proteins were electrophoretically transferred to a Polyvinylidene-difluoride (PVDF) membrane (Sigma-Aldrich) for 1 h at room temperature. After blocking with 5% nonfat dry milk in Tris-buffered saline containing 0.05% Tween 20 (TTBS) for 1 hour at room temperature, the membrane was incubated with rat anti-mouse F4/80 antibody (diluted 1:1000; Abd Serotec) at 4°C overnight with rocking. The membrane was then incubated with secondary anti-rat antibody for 1 h at room temperature. After washing in TTBS, membranes were incubated for one minute in chemiluminescent detection reagent (ECL, GE Healthcare Life Sciences) then exposed to Kodak BioMax MS film (GE Healthcare Life Sciences) for 2 minutes. Western blotting results were quantified using laser densitometry.

### Isolation and culture of human pulmonary artery smooth muscle cells (PA-SMCs) and pulmonary vascular endothelial cells (P-ECs)

Human PA-SMCs were cultured from explants of pulmonary arteries, and P-ECs isolated using immunomagnetic purification were cultured as previously described [27]. Cultures (5·10^4 ^cells/well) of P-ECs and of PA-SMCs were prepared, and IL-6 levels in the culture cell lysates were measured using an ELISA (R&D Systems, Lille, France). Cells were used for the study between passages 3 and 6.

### Effect of IL-6 on human pulmonary artery smooth muscle cells (PA-SMC) migration

PA-SMC migration was assessed using a modified Boyden's chamber (Transwell^®^, Corning Costar Corporation, Badhoevedorp, The Netherlands). The plates were equipped with inserts whose bottoms were sealed with polycarbonate membranes having 6.5 mm internal diameter and 8 μm pore size. The membranes were coated with a solution of 100 μg/ml of type I collagen. Cultured PA-SMCs were trypsinized and suspended at a concentration of 5·10^5 ^cells/ml in DMEM supplemented with 10% fetal calf serum (FCS). PA-SMC suspension, 200 μl, was placed in the upper chamber and allowed to adhere for 24 hours. The medium was then removed and replaced by 200 μl of FCS-free DMEM in the upper chamber and 500 μl of FCS-free DMEM containing IL-6, sIL-6-R, or both (100 ng/ml) in the lower chamber. After 24 h of incubation at 37°C under 5% CO_2_, the cells were fixed and stained using Diff-Quick (Medion Diagnostic, Grafelfing, Germany). The mean number of PA-SMCs from 10 randomly chosen high-power (× 400) fields on the undersurface of the filter was computed.

### Effect of IL-6 on human PA-SMC proliferation

PA-SMCs in DMEM supplemented with 10% FCS were seeded in 24-well plates at a density of 5·10^4 ^cells/well and allowed to adhere. The cells were subjected to 48 h of growth arrest in FCS-free medium then incubated in DMEM with 0.3% FCS supplemented with 0.6 μCi/ml of [^3^H] thymidine with IL-6, sIL-6-R, or both (100 ng/ml of each). After incubation for 24 hours, the cells were washed twice with PBS, exposed to ice-cold 10% trichloroacetic acid, and dissolved in 0.1 N NaOH (0.5 ml/well). [^3^H] thymidine incorporated into the DNA was counted and expressed as counts per minute (cpm) per well.

### Statistical analysis

All results are expressed as mean ± SEM. The nonparametric Mann-Whitney test was used to compare differences between wild-type and IL-6^-/-^normoxic mice. Two-way ANOVA was used to assess the effects, in IL-6^+/+ ^and IL-6^-/- ^mice, of normoxia or hypoxia on hemodynamics, right ventricular hypertrophy, and muscularization as assessed by arterial wall thickness. When ANOVA indicated an interaction between exposure conditions and the genotype, IL-6^+/+ ^and IL-6^-/- ^mice were further compared under each condition using an unpaired nonparametric test. To compare the degree of pulmonary vessel muscularization between the two genotypes under each condition, the nonparametric Mann-Whitney test was used after ordinal classification of pulmonary vessels as nonmuscular, partially muscular, or muscular.

## Results

### Hemodynamic response to acute hypoxia

**Figure 1:**
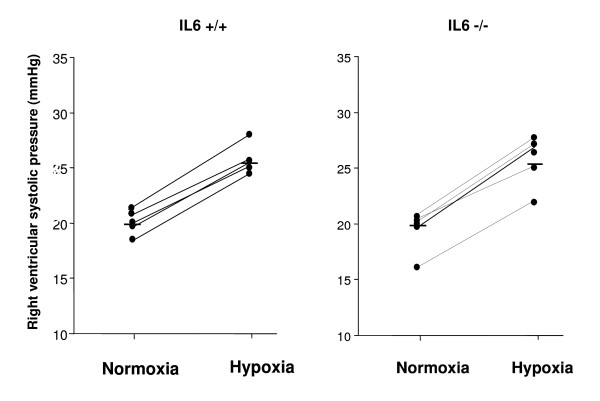
**Hemodynamic response to acute hypoxia in IL-6^+/+ ^and IL-6^-/- ^mice**. Individual and mean (horizontal line) right ventricular systolic pressures (RSVP) in normoxic IL-6^+/+ ^and IL-6^-/- ^mice under ventilation with room air (normoxia) and after 5 min of ventilation with a hypoxic gas mixture (hypoxia). The increase in RVSP induced by acute exposure to 8%O_2 _did not differ between wild-type and IL-6^-/- ^mice.

The effect of an acute hypoxic challenge on RVSP was examined in normoxic mice. Under ventilation with room air, RVSP and heart rate did not differ between IL-6^+/+ ^and IL-6^-/- ^mice. Exposure to 8% O_2 _elicited a large increase in RVSP (Fig. 1), of similar magnitude in IL-6^+/+ ^and IL-6^-/- ^mice, (ΔRVSP, 5.2 ± 0.4 mm Hg, n = 6; vs. 5.7 ± 0.2 mm Hg, n = 5; respectively; NS).

### Lung expression of IL-6, IL-6-R, and gp130 during normoxia and hypoxia

**Figure 2:**
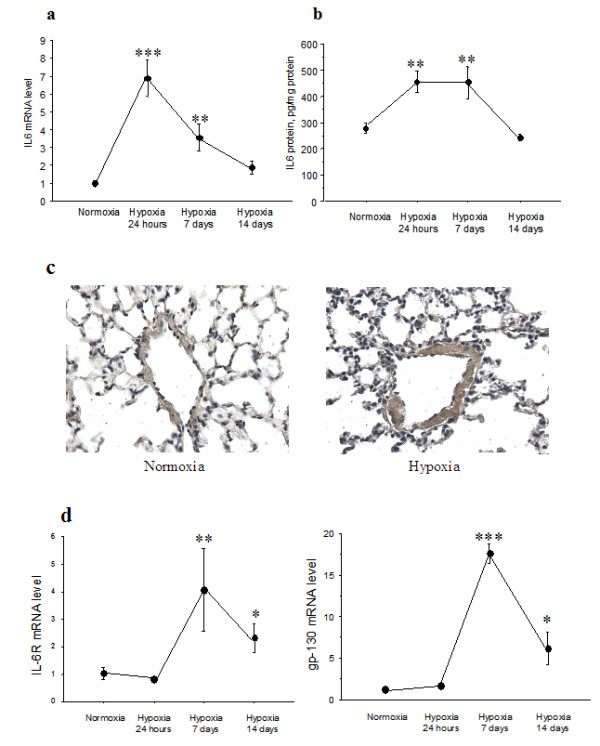
**expression and immunolocalization of interleukin-6 in lungs from IL-6^+/+ ^mice after hypoxia exposure**. IL-6 mRNA levels in total lung tissue determined by real-time quantitative RT-PCR (a) and protein levels assessed by ELISA (b). Each point is the mean ± SEM of at least 8 determinations after exposure to 10% O_2 _for 24 hours, 1 week, or 2 weeks. **P < 0.01, ***P < 0.001 compared with values in normoxic mice. IL-6 immunostaining in lung sections from IL-6^+/+ ^mice under normoxia (c, left panel) and after hypoxia exposure for 7 days (c, right panel). Strong IL-6 immunostaining is visible in vessel walls from the animal exposed to hypoxia (arrows). IL-6R and gp-130 RNA expression in total lung tissue from IL-6^+/+ ^mice exposed to hypoxia (d). Each point is the mean ± SEM of at least 8 determinations after exposure to 10% O_2 _for 24 hours, 1 week, or 2 weeks. *P < 0.05, **P < 0.01, ***P < 0.001 compared to values in normoxic animals.

Exposure to hypoxia was associated with a rapid rise in lung IL-6 mRNA and protein levels in wild-type mice. Lung IL-6 mRNA levels peaked at 24 hours then declined by day 7 and returned to basal values by day 14 (Figure 2a). Lung IL-6 protein levels were also increased at 24 hours but remained elevated on day 7 then returned to basal values by day 14 (Figure 2b). In contrast, levels of lung IL-6 receptor and gp 130 mRNA, which were markedly increased after 1 week of hypoxia, remained elevated after 2 weeks of hypoxia (Figure 2d). Immunohistochemical studies showed IL-6 immunostaining in pulmonary vessel walls from wild-type mice exposed to hypoxia for 7 days (Figure 2c).

### Development of hypoxia-induced pulmonary hypertension and vascular remodeling

**Figure 3:**
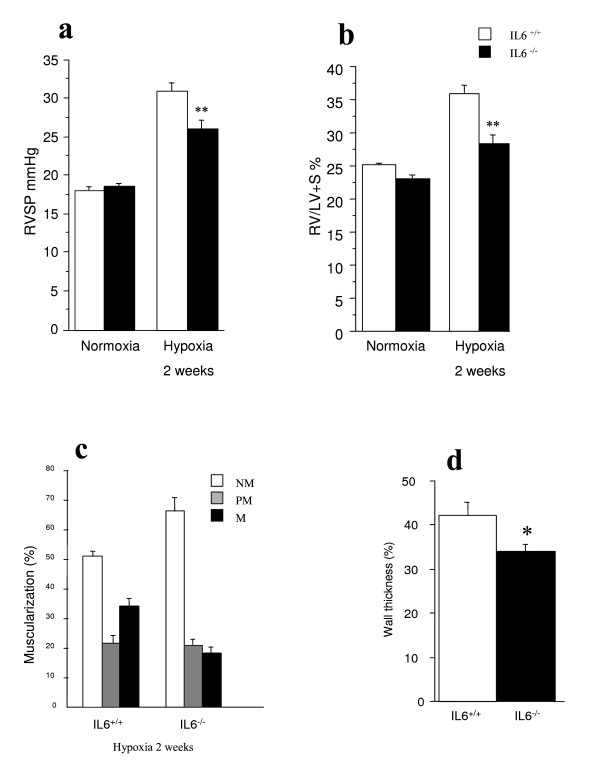
**Development of hypoxic pulmonary hypertension and vascular remodeling in IL-6^+/+ ^and IL-6^-/- ^mice**. Right ventricular systolic pressure (RVSP) (**a**) and weights of the right ventricle/left ventricle + septum (Fulton's index) (**b**) in IL-6^+/+ ^and IL-6^-/- ^mice exposed to normoxia or 10% O_2 _for 2 weeks. **P < 0.01 compared to IL-6^+/+ ^mice under similar conditions. Percentage of muscularized vessels from wild-type IL-6^+/+ ^and IL-6^-/- ^mice (**c**). Twenty to thirty intraacinar vessels were examined in each lung from mice of each genotype after exposure to hypoxia for 2 weeks. Percentages of nonmuscular (NM), partially muscular (PM), and fully muscular (M) vessels differed significantly between IL-6^+/+ ^and after 2 weeks of hypoxia (*P *< 0.05). Normalized wall thickness measured in fully muscular arteries in lungs from IL-6^-/- ^and IL-6^+/+ ^mice exposed to hypoxia for 2 weeks (**d**). *P < 0.05 compared to IL-6^+/+ ^mice exposed to hypoxia for 2 weeks.

Total body weight (BW) was slightly higher in IL6^+/+ ^than in IL6^-/- ^mice (Table 2). Under normoxic conditions, IL6^-/- ^and IL6^+/+ ^mice showed no significant differences in LV weight/BW, RV weight/BW, or heart rate. Exposure to hypoxia was associated with increases in RVSP and Fulton's index in both wild-type and IL6^-/- ^mice. However, after 2 weeks hypoxia, RVSP was significantly lower and right ventricular hypertrophy less severe in IL-6^-/- ^than in IL-6^+/+ ^mice (*P *< 0.01) (fig 3a, b). Furthermore, distal pulmonary vessel muscularization, which also increased with hypoxia exposure, was less marked in IL-6^-/- ^mice than in IL-6^+/+ ^mice, as shown by both the percentage of muscularized pulmonary vessels (Figure 3c) and the wall thickness of muscular arteries (Figure 3d).

**Table 2:** Body weight, heart weight, and hemodynamic data after exposure to 10% O_2 _(hypoxia) or room air (normoxia)

	**Normoxia**		**Hypoxia**	
	IL-6^+/+ ^*n = 8*	IL-6^-/- ^*n = 8*	IL-6^+/+ ^*n = 8*	IL-6^-/- ^*n = 8*

Final body weight (g)	25.2 ± 0.3	22.9 ± 0.5**	24.7 ± 0.6	20.6 ± 0.7**
RV/BW (mg/g)	0.96 ± 0.02	0.85 ± 0.03	1.24 ± 0.07^$$$^	1.02 ± 0.07*
LV/BW (mg/g)	3.8 ± 0.06	3.7 ± 0.15	3.6 ± 0.1	3.9 ± 0.1
Heart rate (beats/min)	308 ± 21	318 ± 24	327 ± 11	318 ± 23

All values are mean ± SEM.

*P < 0.05 and **P < 0.01 compared with corresponding values in wild type mice.

^$^*P *< 0.05 and ^$$^*P *< 0.01 compared with corresponding values under normoxia.

RV/BW, right ventricular weight/body weight; LV/BW, left ventricular weight/body weight.

### Lung macrophage recruitment and cytokine expression during exposure to chronic hypoxia

**Figure 4:**
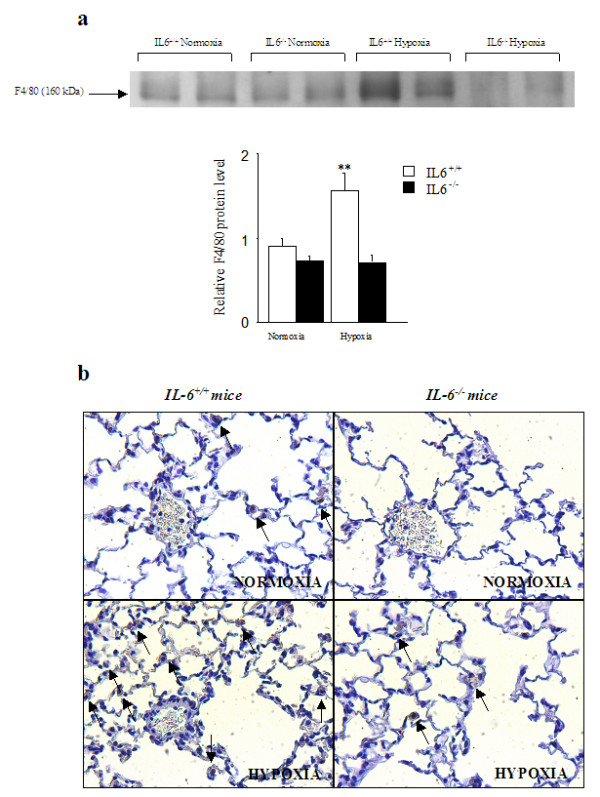
**Lung macrophages recruitment under hypoxic condition in IL-6^+/+ ^and IL-6^-/- ^mice**. Lung F4/80 protein levels assessed by Western blotting in IL-6^+/+ ^mice and IL-6^-/-^mice after normoxia or hypoxia (n = 5 in each group) (**a**). Each bar is the mean ± SEM. *P < 0.05 compared to IL-6^+/+ ^mice exposed to hypoxia of the same duration. Lung macrophage recruitment illustrated by representative photomicrographs showing F4/80 immunostaining in lung sections from IL-6^+/+ ^and IL-6^-/- ^mice under normoxia and hypoxia (**b**). Macrophages are shown by arrows.

**Figure 5:**
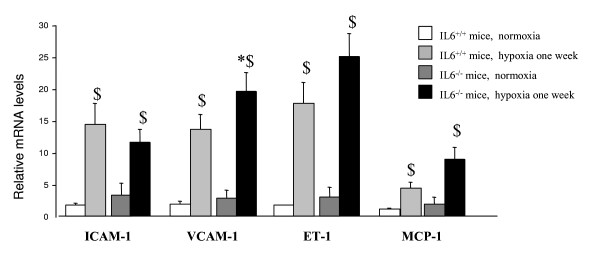
**Lung expression of ICAM-1, VCAM-1, ET-1 and MCP-1 mRNAs in IL-6^-/- ^and IL-6^+/+ ^during normoxic and hypoxic conditions**. Levels of ICAM-1, VCAM-1, ET-1, and MCP-1 mRNAs in lung tissue from IL-6^+/+ ^and IL-6^-/- ^mice after normoxia or 1 week of hypoxia. Each bar is the mean ± SEM (n = 5 in each group). *P < 0.05 and **P < 0.01 compared with corresponding values in wild type mice. ^$^*P *< 0.05 and ^$$^*P *< 0.01 compared with corresponding values under normoxia.

F4/80, a monoclonal antibody that recognizes a murine macrophage-restricted cell surface glycoprotein, has been extensively used to characterize macrophage populations in a wide range of immunological studies [28]. Lung F4/80 protein levels as assessed by Western blotting increased from normoxia to hypoxia in wild-type mice but not in IL-6^-/- ^mice (Figure 4a). Similarly, after hypoxia, lung F4/80 immunostaining was less pronounced in lungs from IL-6^-/- ^mice than from IL-6^+/+ ^mice (Figure 4b). Lung mRNA levels of the inflammatory biomarkers VCAM-1, ICAM-1, and MCP-1, as well as of endothelin-1 (ET-1) were considerably higher after hypoxia than after normoxia, with no differences between IL-6^-/- ^and wild-type mice (Figure 5).

### Growth and migration of PA-SMCs in response to IL-6 and sIL-6-R

**Figure 6:**
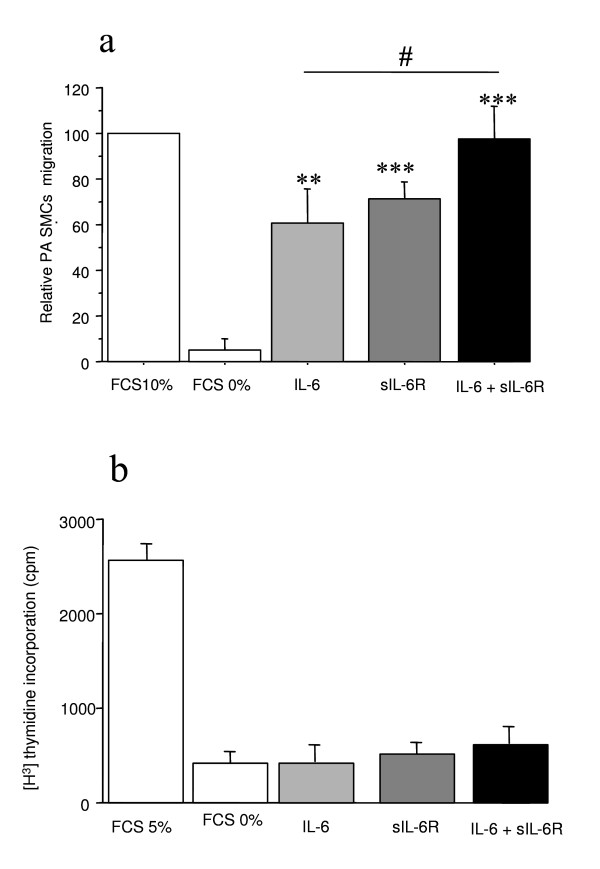
**Effects of IL-6 and its soluble receptor sIL-6-R on migration and proliferation of human pulmonary-artery smooth muscle cells**. Effects of IL-6 and of its soluble receptor sIL-6-R on migration of human pulmonary-artery smooth muscle cells studied using a modified Boyden's chamber (a). The transwell assay demonstrated that both IL-6 and sIL-6R promoted PA-SMC migration and that the effect was stronger when IL-6 and sIL-6R were combined. Each bar is the mean ± SEM for 5 individuals (*P < 0.01, **P < 0.001 vs. basal condition). [^3^H]thymidine incorporation in cultured pulmonary-artery smooth muscle cells (PA-SMCs) from 5 patients (b). The cells were incubated with IL-6, sIL-6R, or both (100 ng/ml of each compound), in the presence of 0.3% fetal calf serum (FCS). Values are the means ± SEM.

We found that IL-6 protein and mRNA levels were considerably higher in quiescent cultured PA-SMCs than in P-ECs (1.5 ± 0.56 vs. 29.3 ± 5 ng/μg protein, *P *< 0.01 and 1.2 ± 0.3 vs. 4.4 ± 1.2 arbitrary units, *P *< 0.05, respectively). Exposure to hypoxia led to a 3-fold increase in IL-6 mRNA levels in PA-SMCs, with a peak after 4 hours' hypoxia exposure (data not shown). Transwell migration assays showed that IL-6 (100 ng/ml) or sIL-6R (100 ng/ml) markedly stimulated human PA-SMC migration. Combining IL-6 and sIL-6R further increased PA-SMC migration (Figure 6a). Treatment of PA-SMCs with IL-6, sIL-6R, or both did not alter [3H]thymidine incorporation into human PA-SMCs (Figure 6b).

## Discussion

The results reported here demonstrate that IL-6 deficiency attenuates the development of hypoxic PH in mice. We found that PH and right ventricular hypertrophy were less severe in IL-6^-/- ^mice than in wild-type mice after 2 weeks of hypoxia. The number of muscular pulmonary vessels was smaller in the IL-6-deficient mice. In contrast, the increase in RVSP elicited by an acute hypoxic challenge was similar in IL-6^-/- ^and IL-6^+/+ ^mice in normoxia. Exposure to hypoxia was associated with a marked increase in lung IL-6 expression, and in vitro studies revealed marked IL-6 synthesis by PA-SMCs with a further increase in response to acute hypoxia. IL-6 markedly stimulated PA-SMC migration without affecting PA-SMC proliferation. Hypoxic IL-6^-/- ^mice showed less inflammatory cell recruitment in the lungs, compared to hypoxic wild-type mice, with no difference in lung expression of adhesion molecules or cytokines. Taken together, these results support a specific role for IL-6 in modulating lung vessel inflammation and remodeling during hypoxic PH progression.

Although strong evidence suggests a role for inflammatory cytokines in the pathogenesis of PH, the involvement of each specific cytokine in pulmonary vascular remodeling remains unclear. Neither do we know how the multifunctional effects of cytokines can, synergistically or independently, affect the processes of inflammation and cell proliferation within lung vessel walls. Here, we focused on IL-6 because we previously found that PH severity in patients with COPD was closely linked to plasma levels and genetic variants of IL-6 [11]. Moreover, circulating IL-6 seems to be increased in most forms of human PH [4,14-16] and several experimental studies recently reported an active role of IL-6 on pulmonary vascular remodeling and hypoxic PH in mice [24]. To assess the specific role for IL-6 in the development of experimental PH, we studied mice exposed to chronic hypoxia. An important finding from our study was that exposure to hypoxia was associated with a marked and early rise in IL-6 mRNA levels, which led to a more prolonged increase in IL-6 protein, lasting up to 7 days but followed by a return to basal levels by day 14. In lung vessels, IL-6 was mainly expressed by SMCs, as shown by immunohistochemical examination of lungs from hypoxic wild-type mice, as well as by studies of cultured cells. We found that IL-6 was expressed by both P-ECs and PA-SMCs but that the amount of IL-6 originating from PA-SMCs was far greater than the amount from P-ECs. Short-term exposure of PA-SMCs to hypoxia also markedly stimulated IL-6 expression, suggesting that PA-SMCs may represent a major source of IL-6 in the lung, especially during the development of hypoxic PH.

These results are consistent with previous reports showing IL-6 induction by hypoxia in cultured vascular cells and prominent IL-6 immunostaining in pulmonary vessels of mice exposed to short-term hypoxia [20]. In these studies, hypoxia induced IL-6 expression via enhanced transcription driven by the nuclear factor IL-6 site in the IL-6 promoter. Thus, exposure to hypoxia leads to a transient rise in IL-6 expression, which does not mimic the sustained IL-6 elevation seen in patients with PH or COPD. The rise in IL-6 protein lasted up to 7 days, and PH developed within 2 weeks in hypoxic mice, allowing us to evaluate whether changes in IL-6 expression affected PH development in our model.

Another point is that the effects of IL-6 on target cells are mediated by plasma membrane receptor complexes containing the IL-6 receptor (which is devoid of transducing activity) and the common signal-transducing receptor chain glycoprotein (gp-130). We found marked increases in hypoxic lung expression of both IL-6 receptor and gp 130, which lasted up to 14 days. Thus, exposure to chronic hypoxia is associated not only with a large increase in lung IL-6 levels, but also with increased expression of the IL-6 receptor.

After 2 weeks of hypoxia, PH was less severe in IL-6-deficient mice than in wild-type littermates. Muscularization of pulmonary arteries after chronic hypoxia was also less marked in IL-6^-/- ^mice than in wild-type mice. These results are consistent with previous reports showing that exogenously administered IL-6 potentiates the development of hypoxic PH in mice [21]. Thus, our results support a role for IL-6 in the development of PH and pulmonary vascular remodeling induced by hypoxia. To investigate whether reduced pulmonary vascular remodeling and PH resulted from decreased pulmonary vasoreactivity to hypoxia, we examined the pulmonary pressure response to acute hypoxia in IL-6^-/- ^and IL-6^+/+^mice. This response, as evaluated based on the RVSP increase, was similar in IL-6^-/- ^and IL-6^+/+ ^normoxic mice. Therefore, the attenuation of PH development and vascular remodeling in the IL-6-deficient mice cannot be explained by decreased pulmonary vasoreactivity to hypoxia. Cytokines have also been shown to affect vascular reactivity in resistance arteries through indirect mechanisms [29]. Cytokines may induce not only vasodilation and hyporesponsiveness to vasoconstrictors, but also constriction mediated by various factors including endothelin-1 and thromboxane A2. Although we did not assess lung prostaglandin synthesis in our mice, an effect mediated by ET-1 was unlikely, given that lung ET-1 levels did not differ between hypoxic IL-6^-/- ^and IL-6^+/+ ^mice. Moreover, IL-6 expression seems increased in many types of human and experimental PH, including monocrotaline-induced PH in rats, suggesting that IL-6 may modulate the extent of PH despite the absence of a hypoxic pulmonary vasoconstrictor component.

The mechanisms by which basal IL-6 levels affect pulmonary vascular remodeling and inflammation remain unclear. IL-6 is a multifunctional cytokine that affects multiple cell types. IL-6 is considered a major cytokine that stimulates vessel-wall cells to express adhesion molecules and chemokines, thus potentiating local inflammatory reactions by stimulating the recruitment of inflammatory cells. In accordance with this view, IL-6^-/- ^mice showed impaired leukocyte accumulation in subcutaneous air pouches, as well as reduced in situ production of chemokines [30]. Another well-known effect of IL-6 stimulation is expression of acute-phase proteins such as C-reactive protein and collagen. On the other hand, recent studies have investigated the potential antiinflammatory effects of IL-6. IL-6 suppressed the generation of the pro-inflammatory cytokines IL-1 and TNF in macrophages exposed to lipopolysaccharide and attenuated the inflammatory response to intratracheally administered lipopolysaccharide [31,32]. Similarly, IL-6 deficiency was recently reported to enhance atherosclerotic lesion formation in ApoE^-/- ^mice [33].

Because alterations in local inflammatory reactions have been reported in IL-6^-/- ^mice [34], we investigated whether the response of IL-6^-/- ^mice to chronic hypoxia differed from that of wild-type mice regarding inflammatory-cell recruitment and expression of adhesion molecules and chemokines in the lung. As expected, our lung F4/80 protein level and immunostaining results indicated decreased macrophage accumulation in lungs from hypoxic IL-6^-/- ^mice compared to wild-type controls. These results are consistent with in vivo stimulation by IL-6 of inflammatory-cell recruitment to sites of inflammation. We did not specifically address the mechanisms by which hypoxia produces this local inflammatory reaction in the lung. However, we found that lung expression of the adhesion molecules ICAM-1 and VCAM-1, and of the cytokine MCP-1, was markedly higher with hypoxia than normoxia in wild-type mice. Surprisingly, similar increases were seen with hypoxia in the IL-6^-/- ^mice, suggesting that the expression of ICAM-1, VCAM-1, and MCP-1 was only partly influenced by IL-6. The increased lung IL-6 levels at the early phase of hypoxia may therefore be viewed as part of an initial whole-lung inflammatory response to hypoxia with a role for IL-6 in mediating inflammatory cell recruitment. The fact that hypoxic PH was less severe in IL-6^-/- ^mice than in wild-type mice despite similar increases in adhesion molecules and cytokines suggests either a specific role for IL-6 in the pulmonary vascular remodeling process or indirect effects mediated via inflammatory cell recruitment.

We therefore investigated whether exogenously added IL-6 affected PA-SMC migration and proliferation and found that IL-6 or its soluble receptor sIL-6R markedly stimulated human PA-SMC migration. Combining IL-6 and its soluble receptor sIL-6R further increased PA-SMC migration. In contrast, treatment of PA-SMCs with IL-6, sIL-6R, or both did not alter [3H]thymidine incorporation into human PA-SMCs. These effects are consistent with the established mediation of IL-6 effects on target cells by plasma membrane receptor complexes containing the IL-6 receptor (devoid of transducing activity) and the common signal transducing receptor chain gp-130 (glycoprotein-130). Whereas the signal transducer element gp-130 is found in many cell types, the IL-6 receptor seems expressed only by cells that respond physiologically to IL-6. Exogenous IL-6 added to human PA-SMCs did not stimulate proliferation even in the presence of sIL-6R. However, IL-6 markedly stimulated PA-SMC migration. These data, together with our finding that exposure to chronic hypoxia is associated with increased lung expression of IL-6R and gp-130 mRNA levels, strongly suggest that IL-6 may act partly as an inducer of PA-SMC migration during chronic hypoxia.

Adding sIL-6R alone stimulated PA-SMC migration, and adding both IL-6 and sIL-6R produced a higher level of stimulation. We interpreted the stimulation induced by sIL-6R alone as an autocrine effect of IL-6 produced by PA-SMCs, an observation also made by others using SMCs from the human aorta [35]. Thus, data obtained with cultured PA-SMCs suggest that PA-SMCs may be physiological targets for IL-6 acting either as a paracrine or as an autocrine factor after being produced by P-ECs or PA-SMCs, respectively.

One conclusion of the present study is that IL-6 can affect both lung inflammation and pulmonary vascular remodeling during exposure to hypoxia. The mechanism by which IL-6 may contribute to vascular remodeling is incompletely understood. IL-6 receptors are expressed not only by inflammatory cells, but also by constitutive vessel-wall cells. Thus, both pulmonary vessel cells and inflammatory cells may be targets for IL-6. Since the hypoxic IL-6^-/- ^mice in our study exhibited decreases in both inflammatory-cell recruitment and vessel-wall remodeling in the lungs, it is unclear whether IL-6 affected pulmonary vascular remodeling by directly targeting vessel-wall cells or by indirect effects mediated by inflammatory cells. Moreover, anti-inflammatory drugs have been shown to affect the early manifestations of acute exposure to hypoxia [36]. The present data and previously published studies therefore suggest that IL-6 may affect vascular remodeling via several mechanisms including a transient effect on vascular permeability [37], a positive effect on inflammatory-cell recruitment [34], and stimulation of vessel-wall remodeling mediated either by direct stimulation of vascular SMC migration or by indirect effects on vascular SMC proliferation

An important limitation of this study is that, in contrast to some types of human PH such as that associated with COPD, or even idiopathic PH, the IL-6 elevation was not sustained. Further studies are therefore needed to elucidate the role for IL-6 and other cytokines that may be synergistically or independently involved in the progression of various forms of human PH associated with lung inflammation.

## Competing interests

The authors declare that they have no competing interests.

## Authors' contributions

LS carried out the experimental work, the data analysis and drafted the manuscript. LT, DR and MI participated in the experimental work. BM participated in the design of the study. SA and SE conceived the hypothesis, advised on experimental work and assisted in drafting the manuscript.
